# Association Between Opium Consumption and Lipid Profile: A Systematic Review and Meta‐Analysis of Observational Studies

**DOI:** 10.1002/hsr2.72851

**Published:** 2026-08-26

**Authors:** Fatemeh Zahra Gafuori Fard, Yaser Mohammadi, Maryam Rezaei, Seyed Mohammad Riahi

**Affiliations:** ^1^ Student Research Committee, School of Medicine Birjand University of Medical Sciences Birjand Iran; ^2^ Student Research Committee Iran University of Medical Sciences Tehran Iran; ^3^ Medical Toxicology & Drug Abuse Research Center Birjand University of Medical Sciences Birjand Iran; ^4^ Department of Epidemiology and Biostatistics, School of Medicine, Cardiovascular Diseases Research Center Birjand University of Medical Sciences Birjand Iran

**Keywords:** addiction, lipid profile, meta‐analysis, opium

## Abstract

**Background:**

Opium, traditionally used for its analgesic and sedative properties, has been widely consumed. Given the conflicting evidence regarding its association with lipid profiles, the present study was conducted to investigate the association between opium consumption and lipid profile parameters.

**Methods:**

A comprehensive search was conducted in PubMed, Scopus, and Web of Science up to May 2025. Search results were screened in two stages by two independent reviewers. Data were extracted and the quality of the included studies was assessed using the JBI checklist. Statistical analysis was performed using STATA‐17.

**Results:**

A total of 36 studies with 5660 opium users and 11,885 controls were included. The meta‐analysis revealed a significant reduction in total cholesterol (WMD = −4.72, 95% CI: −8.72 to −0.71) and HDL‐C (WMD = −1.95, 95% CI: −3.69 to −0.22), while no significant associations were observed for triglycerides, LDL‐C, Apo‐A1, and Apo‐B100. Notably, gender‐based subgroup analysis showed a significant association with lower TG levels in men and lower TC and LDL‐C levels in women. Meta‐regression analyses showed no statistically significant association between duration of opiate use and lipid parameters. Also, overall study quality was high with no evidence of publication bias.

**Conclusion:**

This comprehensive meta‐analysis demonstrates that opium consumption is not associated with a clinically favorable lipid profile. While meta‐regression revealed nonsignificant positive trends between the duration of opium use and lipid parameters, the overall dyslipidemic pattern indicates an unfavorable cardiovascular risk profile. Future longitudinal and multinational studies with standardized exposure assessment are essential to clarify the dose–response relationships and long‐term cardiometabolic consequences of opium use.

## 1 Introduction

Opium has been used for centuries as a natural substance due to its analgesic and sedative properties [1]. In some societies, particularly in the Middle East and South Asia, it is widely consumed and is considered one of the oldest narcotics, posing a serious social and health challenge [2]. One of the primary reasons for the high prevalence of opium use is its easy accessibility and its integration into local traditions and cultural practices in these regions [3]. Additionally, reports indicate that a significant proportion of individuals turn to opium consumption to reduce anxiety, relieve pain, and even due to misconceptions about its benefits [4].

Excessive opium use is associated with various adverse effects, one of the most significant being the development of severe dependency due to its opioid‐like effects [5]. Moreover, opium use is linked to psychological, mental, and physical disorders, including respiratory problems, impaired immune system function, kidney and liver dysfunction [2]. Furthermore, opium abuse can disrupt metabolic pathways, increasing the risk of hypertension, cardiovascular diseases, and type 2 diabetes [6].

One of the most important metabolic aspects of opium use is its effects on lipid metabolism pathways and lipid profiles [7]. Various studies have investigated the impact of opium consumption on lipid profiles; however, their findings are often contradictory and fragmented. Some studies have reported a significant increase in triglycerides and a decrease in HDL‐C levels [8, 9]. Nakhaei and colleagues reported that opium consumption does not provide protective effects against cardiovascular issues and does not improve them [2]. In contrast, some studies have indicated that opium consumption can lead to reductions in certain lipid parameters, such as cholesterol and LDL‐C [1, 10]. For instance, Marmor and colleagues demonstrated that the use of opium or morphine might have favorable effects on cardiovascular diseases [11].

These discrepancies highlight the lack of clarity regarding the exact impact of opium consumption on lipid profiles. On the other hand, abnormalities in lipid profiles directly contribute to the onset and progression of cardiovascular issues [12]. Therefore, identifying and managing factors that influence lipid profiles are of great importance and represent a crucial step in preventing and reducing the burden of cardiovascular diseases.

Given the above considerations highlighting the importance of this social challenge, the present study aims to conduct a systematic review and meta‐analysis to consolidate the existing evidence and provide a comprehensive assessment of the associations between opium consumption and lipid profiles. The findings of this study can help clarify the relationship between opium use and lipid‐related outcomes and provide a broader and more comprehensive perspective for policymakers and experts in various fields.

## 2 Methods

### 2.1 Protocol Registration

The current systematic review and meta‐analysis was registered in Birjand University of Medical Sciences (BUMS) and PROSPERO under Registration Code IR.BUMS.REC.1403.313 and CRD420251078621, respectively. This analysis was conducted in line with Preferred Reporting Items for Systematic Reviews and Meta‐Analyses (PRISMA) guidelines [13].

### 2.2 Search Strategy

To identify relevant articles, a systematic search was conducted across three databases: PubMed, SCOPUS, and Web of Science, up to May 29, 2025. No time, geographic, or language restrictions were applied. The following keywords were used in advanced database searches: (“Triglyceride” OR “Cholesterol” OR “HDL” OR “LDL” OR “Dyslipidemias” OR “Hyperlipidemia” OR “Hypercholesterolemia” OR “Apolipoprotein A‐1” OR “Apolipoprotein B‐100” OR “high density lipoprotein” OR “low density lipoprotein”) AND (“Opium” OR “Morphine” OR “Heroin” OR “opiate*”). The search strategy was developed using Medical Subject Headings (MeSH) terms in PubMed. Additionally, to ensure no studies were missed, the reference lists of the included articles were reviewed. Details of the search queries are reported in Tables S1–S3.

### 2.3 Study Selection

**Figure 1:**
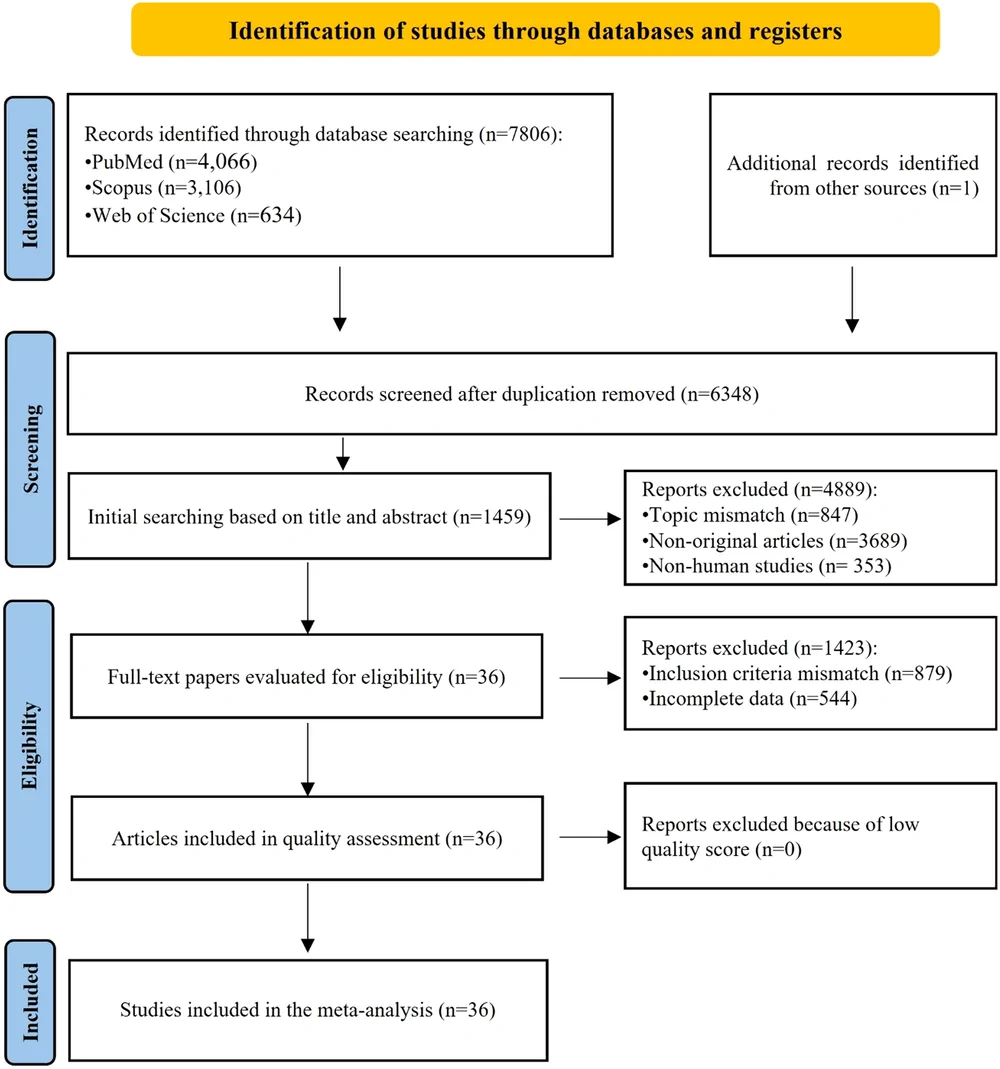
Flowchart showing study selection process.

Search results were imported into EndNote 21 software. Duplicate entries were removed using the “Find Duplicates” tool. Subsequently, articles were screened independently by two researchers (F. G. and Y. M.), first by reviewing titles and abstracts, and then by examining the full texts. Any disagreements were resolved through discussion with a third reviewer (S. M. R.). Finally, eligible articles for inclusion in the systematic review and meta‐analysis were selected based on the inclusion and exclusion criteria agreed upon by the three independent reviewers. Figure 1 illustrates the article selection process.

### 2.4 Inclusion and Exclusion Criteria

Inclusion Criteria: (1) Case‐control studies, cross‐sectional studies, and cohort studies. (2) Studies assessing outcomes of interest related to the lipid profile, including the effects of opium on triglycerides (TG), cholesterol, high‐density lipoprotein‐cholesterol (HDL‐C), low‐density lipoprotein‐cholesterol (LDL‐C), apolipoprotein A‐1, and apolipoprotein B‐100.

Exclusion Criteria: (1) Studies without outcomes related to the lipid profile. (2) Participants using unknown substances. (3) Animal studies, case reports, conference abstracts, review articles, and letters.

### 2.5 PECOS

To clarify the search question, the PECOS (Population, Exposure, Comparator, Outcome, and Study design) was used. Population: Adults. Exposure: Opium user, Control/Comparison: Opium nonuser. Outcome: Lipid profile levels (TG, cholesterol, HDL‐C, LDL‐C, Apolipoprotein A‐1, and Apolipoprotein B‐100). Study design: Cross‐sectional, case‐control Studies, and cohort studies.

### 2.6 Data Extraction

Two researchers (F. G. and Y. M.) independently extracted data from the included studies, and the extracted data were subsequently reviewed by an additional researcher (S. M. R.). The extracted information encompassed the following details: first author, year of publication, country, type of substance consumption, study design, age, gender, sample size, underlying conditions, duration of consumption, and lipid profile parameters (TG, cholesterol, HDL‐C, LDL‐C, apolipoprotein A‐1, and apolipoprotein B‐100).

### 2.7 Quality Evaluation

The included articles were evaluated by using Joanna Briggs Institute (JBI) Critical Appraisal checklist which have eight evaluation criteria: (1) appropriate inclusion criteria; (2) description of the study subject and setting; (3) valid measurement for all participants; (4) standard criteria for measuring the condition; (5) identification of confounding factors; (6) use strategies to deal with confounding factors; (7) valid outcome measurement; and (8) appropriate statistical analysis. Each question should be answered as “Yes,” “No,” “Unclear,” or “Not applicable.” According to the total quality score, each article was scored as high quality (85% and above), moderate (60%–85% score), or low quality (60% score). Finally, the articles with high quality score (*n* = 36) were included in the analysis. The quality assessment process was conducted by two independent reviewers (F. G. and Y. M.) and reviewed by another researcher (S. M. R.).

### 2.8 Statistical Analysis

The statistical analyses were performed using Stata software version 11 (StataCorp, College Station, TX, USA). We evaluated the association between opium consumption and lipid profile parameters using the weighted mean difference (WMD), defined as the pooled difference in mean lipid values between opium users and controls, along with a 95% confidence interval (CI) [14]. In the data analysis, lipid profile values in the case group (opium users) were subtracted from those in the control group (WMD = case − control). Accordingly, a negative WMD indicates lower lipid levels among opium users, whereas a positive WMD indicates higher lipid levels compared with controls. Statistical significance was interpreted based on the 95% CI, such that if the CI included zero, the association was considered statistically nonsignificant; otherwise, it was considered statistically significant [15]. Between‐study heterogeneity, defined as variability beyond chance, was assessed using Cochran's *Q* test and quantified using the *I* ^2^ statistic, which represents the percentage of total variation attributable to heterogeneity rather than sampling error. *I* ^2^ values exceeding 60% were considered indicative of substantial heterogeneity, consistent with established methodological guidelines [16, 17, 18, 19]. To address heterogeneity, prespecified subgroup analyses were conducted according to gender, age (years), study population, country, matching factors, study design, duration of opium use, and type of substance consumed, based on biological plausibility and prior evidence. In addition, meta‐regression analyses were performed as exploratory analyses to examine the potential modifying effect of continuous or study‐level covariates, including duration of opium consumption, on lipid outcomes. Finally, publication bias, defined as selective reporting of statistically significant results, was evaluated using Egger's regression asymmetry test, with a *p* < 0.10 considered suggestive of small‐study effects [19, 20].

## 3 Results

### 3.1 Search Results and Study Characteristics

Following a systematic search and a two‐stage screening process, 36 studies (cross‐sectional = 27; case‐control = 9, cohort = 0) meeting the inclusion criteria were included in the meta‐analysis. The studies were published between 1983 and 2022. A total of 5660 individuals with substance use and 11,885 healthy individuals were analyzed. A majority of the studies (83%) were conducted in Iran (*n* = 30), 5% in Italy (*n* = 2), and the remaining studies were conducted in Afghanistan, Turkey, Pakistan, and Yugoslavia (*n* = 1 each). Among the included studies, 87% reported opium use (*n* = 31), 13% reported heroin use (*n* = 5). The reported duration of addiction ranged from 1 to 208 months. Tables 1 and 2 show the characteristics of the included studies.

**Table 1:** Characteristics of the included studies (cross‐sectional) in the meta‐analysis.

First author (publication)	Country	Type of substance	*N* case (male%)	*N* control (male %)	Duration (month)	Matching	Underlying disease	Results (addicts compared to nonaddict)						Quality score (%)
								TG	TC	LDL‐C	HDL‐C	Apo A‐1	Apo B‐100	
Onikzeh et al. [21]	Iran	Opium	40 (85)	55 (47.3)	NA	NA	Postangiography	↓	↔	↔	↔	NA	NA	100
Bahrami et al. [22]	Iran	Opium	59 (100)	59 (100)	NA	Age, BMI, sex	CAD, CAE	↔	↔	↔	↓	NA	NA	87/5
Moezi et al. [1]	Iran	Opium	324 (73.5)	401 (59.1)	NA	Age, BMI, duration of diabetes, cigarette smoking, medication, education	Candidate for coronary angiography	↓	↔	↔	↔	NA	NA	100
Kazemi et al. [23]	Iran	Opium	2239 (96)	7061 (30)	NA	NA	None	↔	↓	↓	↓	NA	NA	100
Hamrah et al. [24]	Afghanistan	Opium	161 (84.5)	413 (34.1)	NA	NA	None	↔	↓	NA	NA	NA	NA	100
Hoseini et al. [25]	Iran	Opium	45 (44.4)	135 (35.6)	100.44 ± 69.84	Age, sex	DM2	↑	↔	↔	↔	NA	NA	100
Barzehkar et al. [26]	Iran	Opium	40 (85)	40 (85)	24	NA	None	↔	↑	↑	↔	NA	NA	100
Aghadavoudi et al. [8]	Iran	Opium	117 (96.58)	208 (23.89)	151.2 ± 92.4	NA	CABG	↑	↔	↑	NA	NA	NA	100
Masoomi et al. [27]	Iran	Opium	103 (100)	114 (100)	1	NA	CAD	↔	↔	NA	NA	NA	NA	100
Afarinesh et al. [28]	Iran	Opium	89 (100)	110 (100)	24	Age, education	None	↔	↔	NA	NA	NA	NA	100
Sanli et al. [29]	Turkey	Opium	46 (97.5)	69 (92.8)	NA	NA	None	↔	↔	↔	↔	NA	NA	100
Bayani et al. [30]	Iran	Opium	48 (45.8)	49 (24.5)	6	Age, Body Mass Index (BMI), diabetes duration	DM2 admitted to CCU	↔	↔	↔	↔	NA	NA	87/5
Javadi et al. [31]	Iran	Opium	152 (80)	152 (68.2)	NA	NA	AMI	↔	↔	↔	↔	NA	NA	100
Rahimi et al. [32]	Iran	Opium	179 (56.4)	195 (15.9)	3	NA	DM2	↔	↔	↔	↓	NA	NA	100
Divsalar et al. [10]	Iran	Heroin	70 (100)	100 (100)	24	Age, sex, body mass index, educational level	None	↔	↓	NA	NA	NA	NA	100
Qasim et al. [7]	Pakistan	Opium	50 (100)	25 (100)	120	age	Psychologic patient	↓	↑	NA	↑	NA	NA	75
Roohafza et al. [33]	Iran	Opium	126 (100)	126 (100)	208.8 ± 124.8	Age, smoking status	AMI	↔	↔	↔	↔	NA	NA	100
Dehghani et al. [34]	Iran	Opium	239 (86.6)	221 (64.1)	NA	NA	AMI	↔	↔	↔	↔	NA	NA	87/5
Najalfi and Sheikhvatan [35]	Iran	Opium	26 (96.2)	206 (58.3)	NA	NA	DM2, CAD, candidate CABG	↔	↔	↔	↔	NA	NA	100
Saadat et al. [36]	Iran	Opium	15 (100)	15 (100)	NA	Dyslipidemia, hypertension	CAD	NA	NA	↔	NA	NA	NA	75
Pereska et al. [9]	Yugoslavia	Heroin	66 (NA)	25 (NA)	12	Age, BMI, sex	None	↑	↓	↔	↓	↓	↓	100
Hosseini et al. [37]	Iran	Opium	228 (92.1)	228 (92.1)	3	Age, sex, smoking status	DM2	↓	↔	↔	↔	NA	NA	100
Rezvanfar et al. [38]	Iran	Opium	88 (100)	144 (100)	36 ± 6	NA	DM2	↓	↔	↔	↔	NA	NA	100
Kouros et al. [39]	Iran	Opium	42 (100)	35 (100)	9.5 ± 1.7	Age, sex, body mass index (BMI), educational level	None	↔	↓	NA	NA	NA	NA	100
Kouros et al. [39]	Iran	Heroin	35 (100)	35 (100)	8.9 ± 2	Age, sex, body mass index (BMI), educational level	None	↔	↓	NA	NA	NA	NA	100
Shirani et al. [6]	Iran	Opium	148 (100)	791 (100)	NA	NA	Candidate CABG	↔	↔	↔	↔	NA	NA	100
Masoomi et al. [40]	Iran	Opium	126 (89.7)	114 (71.9)	NA	NA	CAD	↓	↔	↔	↔	NA	NA	100
Davoodi et al. [41]	Iran	Opium	45 (100)	115 (100)	NA	Age	AMI	↓	↔	↔	↔	NA	NA	100

Abbreviations: DM2, diabetes mellitus type 2; CABG, coronary artery bypass graft; AMI, acute myocardial infarction; CAD, coronary artery disease; CAE, coronary artery ectasia; NA, not available.

**Table 2:** Characteristics of the included studies (case‐control) in the meta‐analysis.

First author (publication)	Country	Type of substance	*n* case (male%)	*n* control (male%)	Duration (month)	Matching	Underlying disease	Results (addicts compared to nonaddict)					Quality score (%)
								TG	TC	LDL‐C	HDL‐C	Apo A‐1		Apo B‐100
Kahnoji and Najmaei [42]	Iran	Opium	39 (51.3)	40 (56.97)	NA	NA	CAD	↔	↑	↑	↓	NA	NA	87/5
Montazerifar et al. [43]	Iran	Heroin	25 (80)	22 (54.54)	NA	age	None	↑	↔	↔	↔	NA	NA	90
Azod et al. [44]	Iran	Opium	23 (100)	46 (100)	NA	Age, BMI, duration of diabetes, cigarette smoking, medication, education	DM2	↔	NA	↔	↔	NA	NA	100
Fatemi et al. [45]	Iran	Opium	100 (100)	75 (100)	NA	Age, sex	None	↔	↓	↔	↔	NA	NA	90
Asgary et al. [46]	Iran	Opium	360 (100)	360 (100)	NA	Age, smoked cigarette/day	None	↔	↔	↔	↓	↓	↑	100
Karam et al. [47]	Iran	Opium	49 (46.93)	49 (46.93)	12	Age, BMI, latest fasting glucose, cigarette smoking, medications	None	↔	↔	NA	↓	NA	NA	100
Asgary et al. [48]	Iran	Opium	32 (100)	32 (100)	107.04 ± 60	NA	None	↔	↔	↔	↔	NA	NA	100
Maccari et al. [49]	Italy	Heroin	66 (NA)	23 (NA)	NA	Age	None	↑	↓	NA	↓	↔	↔	70
Ceriello et al. [50]	Italy	Opium	20 (100)	20 (100)	NA	Age, weight	None	NA	NA	NA	↓	↓	NA	70

Abbreviations: AMI, acute myocardial infarction; CAD, coronary artery disease; CAE, coronary artery ectasia; CABG, coronary artery bypass graft; DM2, diabetes mellitus type 2; NA, not available.

Overall, subgroup analysis was performed in Stata software version 11. Subgroup analysis was included of gender (male, female, both), age (< 50 years, > 50 years), publication of study (diabetes, heart disease, healthy), country (Iran, other), matching factors (nonmatching, full matching), type of study (case control, cross sectional), duration (< 12 months, > 12 months), and type of substance (opium, heroin).

### 3.2 Meta‐Analysis Results

#### 3.2.1 Triglyceride (TG)

The overall results, including 31 articles, 5433 cases, and 11,628 controls, showed a nonsignificant reduction in triglyceride levels associated with opium consumption on TG levels (WMD = −2.79, 95% CI: −7.20 to 1.61) (Table 3). However, the high *I* ^2^ value (79.6%) suggested substantial heterogeneity across the included studies, highlighting the necessity of subgroup analyses.

**Table 3:** Overall, and subgroup analysis for determining the effect of opium on triglycerides (TG).

Variables		Articles (*n*)	Case (*n*)	Control (*n*)	WMD (95% CI)	*p*_Egger_	_*I*_2
	Total	31	5433	11,628	−2.79 (−7.20 to 1.61)	0.427	79.6
Gender	Male	15	1549	2322	−7.72 (−15.08 to −0.36)	0.169	82.7
	Female	2	112	190	−1.72 (−19.41 to 15.97)	—	43.7
	Both	19	4215	9666	0.03 (−5.99 to 6.05)	0.977	74.7
Age (year)	< 50	8	2916	7721	−2.00 (−9.83 to 5.82)	0.842	79.9
	> 50	17	2131	3593	−8.36 (−19.30 to 2.58)	0.136	83.9
Population of study	Diabetes	5	589	751	−11.45 (−35.57 to 12.67)	0.401	88.0
	Heart disease	13	1533	2592	−6.63 (−20.13 to 6.87)	0.102	82.2
	Healthy	13	3311	8285	−0.83 (−5.15 to 3.49)	0.999	73.9
Country	Iran	26	5044	11,073	−5.99 (−11.29 to −0.69)	0.180	80.3
	Other	5	389	555	7.68 (−0.62 to 15.97)	0.376	67.9
Matching factors	Nonmatching	15	3735	9815	−4.65 (−14.05 to 4.75)	0.563	83.6
	Full matching	16	1698	1813	−2.87 (−8.28 to 2.53)	0.565	75.6
Type of study	Case control	6	433	614	4.73 (−4.34 to 13.81)	0.483	61.0
	Cross sectional	25	5000	11,014	−4.97 (−10.13 to 0.18)	0.240	81.9
Duration	< 12	4	305	314	−3.83 (−14.69 to 7.03)	0.288	48.3
	> 12	10	889	1082	−1.39 (−10.48 to 7.71)	0.871	81.5
Substance type	Opium	27	5241	11,523	−4.29 (−9.15 to 0.57)	0.242	79.7
	Heroin	4	192	105	8.17 (−10.29 to 26.62)	0.690	78.2

In the subgroup analyses, varied results were observed. Within the male subgroup, opium consumption was associated with a statistically significant reduction in TG levels (WMD = −7.72, 95% CI: −15.08 to −0.36; *p* = 0.03). In contrast, this effect was not significant in females or in studies that included both genders.

Geographically, studies conducted in Iran reported a significant decrease (WMD: −5.99 mg/dL; 95% CI: −11.29 to −0.69), while studies from other countries showed a nonsignificant increase. Finally, examination of the substance type showed that opium, in general, was associated with a nonsignificant reduction in TG, while heroin indicated a nonsignificant increase, though this subgroup comprised only four articles. Further analysis based on the age, study population, matching factors, type of study, and duration also indicated nonsignificant reductions in TG. Table 3 shows the overall and subgroup analysis of opium consumption on TG levels.

#### 3.2.2 Low‐Density Lipoprotein‐Cholesterol (LDL‐C)

This comprehensive meta‐analysis, integrating data from 25 articles, 4876 cases, and 10,885 controls, investigated the overall and subgroup effects of opium consumption on LDL‐C levels. The overarching analysis revealed no statistically significant association between opium use and LDL‐C levels (WMD = 0.79, 95% CI: −5.07 to 6.67), with a high degree of heterogeneity observed across the studies (*I* ^2^ = 96.1%) (Table 4). This substantial heterogeneity underscores the importance of examining the effects within specific subgroups.

**Table 4:** Overall, and subgroup analysis for determining the effect of opium on low‐density lipoprotein‐cholesterol (LDL‐C).

Variables		Articles (*n*)	Case (*n*)	Control (*n*)	WMD (95% CI)	^*p*^_Egger_	_*I*_2
	Total	25	4876	10,885	0.79 (−5.07 to 6.67)	0.947	96.1
Gender	Male	10	1175	1941	−3.94 (−10.16 to 2.29)	0.067	88.7
	Female	1	86	164	−10.47 (−18.47 to −2.472)	—	—
	Both	16	3939	9181	3.48 (−2.47 to 9.42)	0.420	92.4
Age (year)	< 50	5	2736	7537	−3.59 (−18.17 to 10.99)	0.653	98.8
	> 50	17	1993	3226	2.59 (−5.17 to 10.35)	0.225	94.3
Population of study	Diabetes	5	563	748	2.37 (−2.99 to 7.72)	0.343	44.1
	Heart disease	13	1445	2493	2.94 (−7.16 to 13.04)	0.303	95.6
	Healthy	7	2868	7644	−5.23 (−17.55 to 7.09)	0.543	98.2
Country	Iran	23	4764	10,791	0.79 (−5.53 to 7.12)	0.965	96.3
	Other	2	112	94	0.67 (−15.19 to 16.54)	—	91.5
Matching factors	Nonmatching	13	3471	9288	3.20 (−4.87 to 11.26)	0.844	96.3
	Full matching	12	1405	1597	−2.35 (−8.74 to 4.05)	0.062	87.5
Type of study	Case control	4	180	175	−4.71 (−16.04 to 6.63)	0.741	49.0
	Cross sectional	21	4696	10,710	1.62 (−4.75 to 7.99)	0.863	96.7
Duration	< 12 m	1	179	195	8.20 (0.55 to 15.854)	—	—
	> 12 m	8	750	947	3.71 (−11.05 to 18.47)	0.627	96.0
Substance type	Opium	23	4785	10,838	1.57 (−4.66 to 7.81)	0.868	96.4
	Heroin	2	91	47	−7.47 (−12.63 to −2.31)	—	0.0

Subgroup analysis based on gender yielded intriguing results. While there was a nonsignificant decrease in LDL‐C among males (WMD = −3.94, 95% CI: −10.16 to 2.29), a single study on females reported a notable decrease in LDL‐C (WMD = −10.47, 95% CI: −18.47 to −2.472). Studies combining both genders showed a nonsignificant increase in LDL‐C (WMD = 3.48, 95% CI: −2.47 to 9.42). Geographically, studies conducted in Iran showed a nonsignificant increase in LDL‐C, while studies from other countries, based on a limited number of articles, also did not show a clear trend. Regarding the duration of opium consumption, a single study for durations less than 12 months indicated a significant increase in LDL‐C, while longer durations (> 12 months) showed a nonsignificant increase. Lastly, concerning the substance type, opium itself demonstrated a nonsignificant increase in LDL‐C, whereas heroin, based on two articles, suggested a significant decrease. Further examination by the age, population of study, matching factors, and type of study similarly did not yield any change on LDL‐C level. Table 4 shows the overall and subgroup analysis of opium use on LDL‐C levels.

#### 3.2.3 Total Cholesterol (TC)

The results of the analysis of 31 articles, 5433 cases, and 11,628 controls showed a significant reduction in TC levels associated with opium use (WMD = −4.72, 95% CI: −8.72 to −0.71) (Table 5). However, the high *I* ^2^ value of 93.5% signifies substantial heterogeneity across the included studies, necessitating a detailed examination of subgroup effects.

**Table 5:** Overall, and subgroup analysis for determining the effect of opium on total cholesterol (TC).

Variables		Articles (*n*)	Case (*n*)	Control (*n*)	WMD (95% CI)	^*p*^_Egger_	_*I*_2
	Total	31	5433	11,628	−4.72 (−8.72 to −0.71)	0.185	93.5
Gender	Male	15	1549	2322	−4.85 (−9.79 to 0.04)	0.676	91.9
	Female	2	112	190	−14.20 (−22.88 to −5.53)	—	0.00
	both	19	4215	9666	−5.29 (−12.24 to 1.67)	0.073	92.5
Age (year)	< 50	8	2916	7721	−2.05 (−9.13 to 5.03)	0.006	90.3
	> 50	17	2131	3593	−5.21 (−9.71 to −0.70)	0.084	78.4
Population of study	Diabetes	5	589	751	−6.47 (−11.18 to −1.77)	0.168	18.8
	Heart disease	13	1533	2592	−3.52 (−7.51 to 0.47)	0.094	53.6
	Healthy	13	3311	8285	−4.95 (−12.78 to 2.89)	0.606	97.2
Country	Iran	26	5044	11,073	−4.47 (−8.52 to −0.42)	0.225	92.9
	Other	5	389	555	−2.66 (−21.49 to 16.18)	0.307	94.8
Matching factors	Nonmatching	15	3735	9815	−4.41 (−10.04 to 1.22)	0.076	92.7
	Full matching	16	1698	1813	−4.99 (−10.65 to 0.65)	0.932	90.0
Type of study	Case control	6	433	614	−16.81 (−24.97 to −8.64)	0.549	64.8
	Cross sectional	25	5000	11,014	−2.19 (−6.35 to 1.96)	0.518	93.6
Duration	< 12 m	4	305	314	−13.03 (−22.39 to −3.67)	0.145	56.7
	> 12 m	10	889	1082	−1.06 (−7.77 to 5.65)	0.237	82.0
Substance type	Opium	27	5241	11,523	−3.37 (−7.53 to 0.78)	0.344	93.9
	Heroin	4	192	105	−15.09 (−32.43 to 2.24)	0.726	84.6

Subgroup analysis based on gender revealed distinct patterns. While males showed a nonsignificant decrease in TC (WMD = −4.85, 95% CI: −9.79 to 0.04), a significant decrease was observed in females (WMD = −14.20, 95% CI: −22.88 to −5.53). Studies combining both genders also showed a nonsignificant decrease (WMD = −5.29, 95% CI: −12.24 to 1.67). Analysis of the data of participants over 50 years showed a statistically significant reduction in TC (WMD = −5.21; 95% CI: −9.71 to −0.70; *I* ^2^ = 78.4%).

Further stratification by population of study indicated significant decreases in TC across diabetic populations (WMD = −6.47; 95% CI: −11.18 to −1.77) while reduction in heart disease, and healthy populations were nonsignificant. Geographically, studies from Iran reported a significant decrease in TC (WMD = −4.47; 95% CI: −8.52 to −0.42; *I* ^2^ = 92.9%). Case‐control studies showed a strong and significant reduction in TC level (WMD = −16.81; 95% CI: −24.97 to −8.64) while cross‐sectional studies did not show significant differences (WMD = −2.19; 95% CI: −6.35 to 1.96). Studies with less than 12 months of use reported a significant decrease in TC level (WMD = −13.03; 95% CI: −22.39 to −3.67). Analysis in subgroup of substance type and matching factors showed no change in TC level. Table 5 shows the overall and subgroup analysis of opium use on TC levels.

#### 3.2.4 High‐Density Lipoprotein‐Cholesterol (HDL‐C)

Analysis of data from 26 articles, 4906 cases, and 10,733 controls, showed a significant reduction in HDL‐C levels associated with opium use (WMD = −1.95, 95% CI: −3.69 to −0.22), with a very high degree of heterogeneity observed across the studies (*I* ^2^ = 95.3%) (Table 6). This substantial heterogeneity necessitates a detailed exploration of the effects within specific subgroups.

**Table 6:** Overall, and subgroup analysis for determining the effect of opium on high‐density lipoprotein‐cholesterol (HDL‐C).

Variables		Articles (*n*)	Case (*n*)	Control (*n*)	WMD (95% CI)	^*p*^_Egger_	_*I*_2
	Total	26	4906	10,733	−1.95 (−3.69 to −0.22)	0.184	95.3
Gender	Male	11	1230	1948	−1.61 (−3.04 to −0.17)	0.996	84.6
	Female	2	112	190	−0.62 (−3.18 to 1.95)	—	0.0
	Both	17	3937	9045	−2.04 (−4.87 to 0.79)	0.098	96.0
Age (year)	< 50	6	2756	7557	−3.55 (−8.73 to 1.63)	0.517	98.4
	> 50	15	1853	2972	−0.79 (−2.19 to 0.59)	0.767	83.7
Population of study	Diabetes	5	589	751	−2.70 (−7.47 to 2.06)	0.970	93.8
	Heart disease	11	1313	2270	−0.64 (−1.16 to −0.13)	0.086	0.0
	Healthy	10	3004	7712	−4.24 (−8.36 to −0.13)	0.228	97.9
Country	Iran	21	4658	10,571	−0.55 (−2.24 to 1.14)	0.628	94.7
	Other	5	248	162	−8.72 (−17.65 to 0.22)	0.187	96.6
Matching factors	Nonmatching	12	3354	9080	−0.77 (−3.41 to 1.87)	0.303	96.2
	Full matching	14	1552	1653	−2.99 (−5.21 to −0.77)	0.788	91.7
Type of study	Case control	6	292	221	−6.84 (−14.29 to 0.61)	0.584	94.9
	Cross sectional	20	4614	10,512	−0.68 (−2.43 to 1.06)	0.573	95.1
Duration	< 12 m	2	228	244	−6.61 (−16.01 to 2.78)	—	92.5
	> 12 m	8	683	764	−0.51 (−2.04 to 1.02)	0.463	57.3
Substance type	Opium	23	4749	10,663	−1.04 (−2.70 to 0.61)	0.416	94.7
	Heroin	3	157	70	−9.31 (−21.14 to 2.53)	0.727	95.6

Subgroup analysis based on gender showed significant decreases in HDL‐C for males (WMD = −1.61, 95% CI: −3.04 to −0.17) and nonsignificant changes in females (WMD = −0.62, 95% CI: −3.18 to 1.95) and both gender subgroups (WMD = −2.04, 95% CI: −4.87 to 0.79).

Further examination by population of study revealed nonsignificant decreases in HDL‐C across diabetic population. However, for the heart disease population, a significant decrease was observed (WMD = −0.64, 95% CI: −1.16 to −0.13), though its statistical significance (*p* = 0.086) was marginal and the *I* ^2^ was notably low (0.0%), suggesting homogeneity within this specific subgroup. Also in healthy population, a significant decrease was reported (WMD = −4.24; 95% CI: −8.36 to −0.13). Fully matched studies reported a significant decrease notable (WMD = −2.99; 95% CI: −5.21 to −0.77). Further analysis based on the age, country, type of study, duration, and substance type also indicated no change in HDL‐C level. Table 6 shows the overall and subgroup analysis of opium consumption on HDL‐C levels.

#### 3.2.5 Apo‐Lipoprotein A1 and B100

The results indicated that opium use might lead to a nonsignificant decrease in apo‐lipoprotein A1 levels (WMD = − 24.27, 95% CI: −51.79 to 3.25), while concurrently causing a nonsignificant increase in apo‐lipoprotein B100 levels (WMD = 3.15, 95% CI: −23.68 to 29.98). Table 7 shows the overall analysis of opium use on apo‐lipoprotein levels.

**Table 7:** Overall, and subgroup analysis for determining effect of opium on types of Apo‐lipoprotein.

Variables	Articles (*n*)	Case (*n*)	Control (*n*)	WMD (95% CI)	^*p*^_Egger_	_*I*_^2^
Apo‐lipoprotein A1	4	512	428	−24.27 (−51.79 to 3.25)	0.680	99.1
Apo‐lipoprotein B100	3	492	408	3.15 (−23.68 to 29.98)	0.852	99.8

### 3.3 Meta‐Regression Analysis

**Figure 2:**
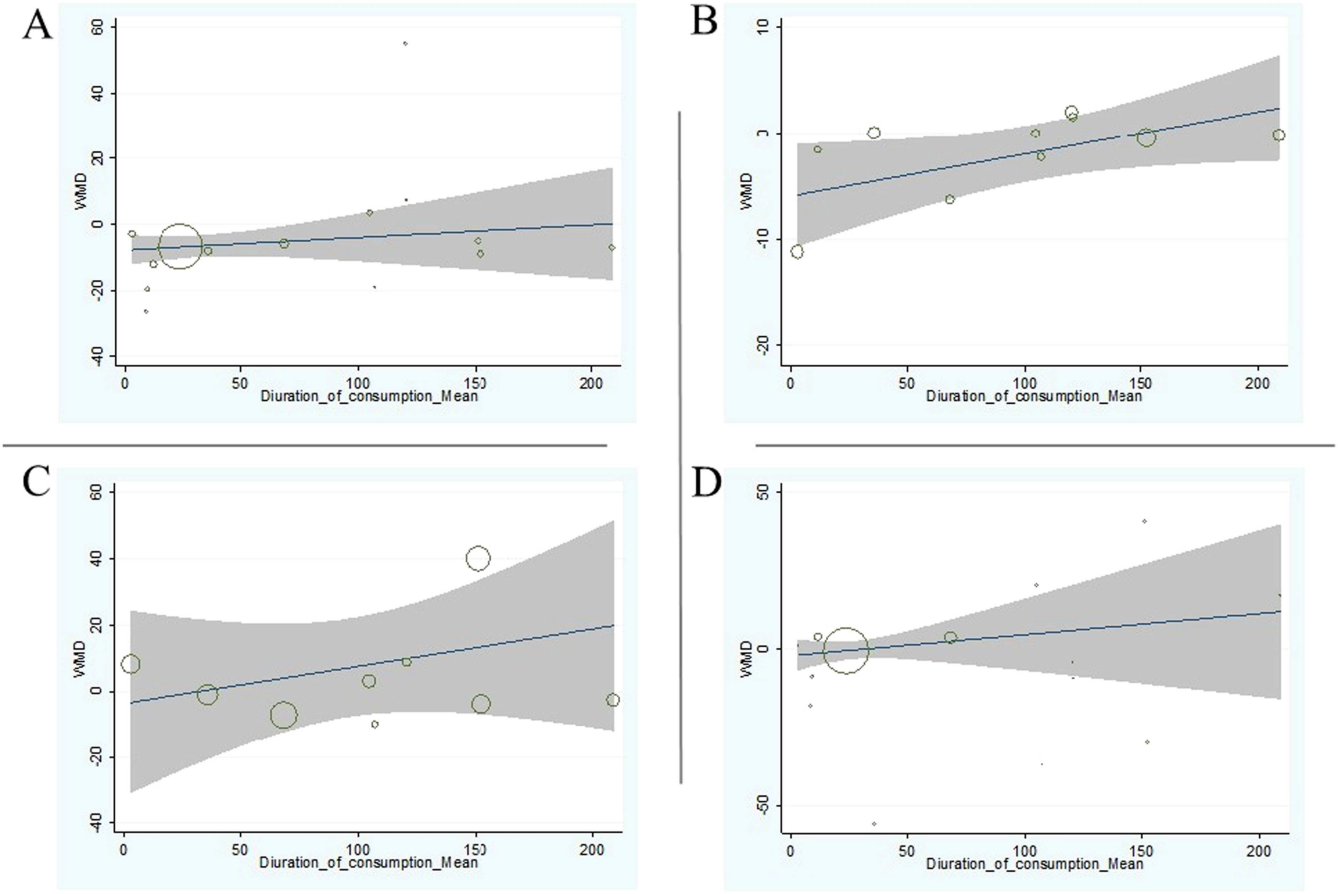
Meta‐Regression analysis of opium consumption duration on lipid profiles. (A) TC: total cholesterol (*β* = +0.04, *p* = 0.41), (B) HDL‐C: high‐density lipoprotein‐cholesterol (*β* = +0.03, P 0.28), (C) LDL‐C: low‐density lipoprotein‐cholesterol (*β* = +0.06, *p* = 0.19), and (D) TG: triglyceride (*β* = +0.05, *p* = 0.46).

Figure 2 presents the results of meta‐regression analyses investigating the relationship between the mean duration of opium consumption and changes in total cholesterol (TC), HDL‐C, LDL‐C, and triglycerides (TG). While subtle trends were observed across the lipid parameters, none of these linear relationships reached statistical significance. Specifically, for TC (Figure 2A), HDL‐C (Figure 2B), LDL‐C (Figure 2C), and TG (Figure 2D), the WMD generally trended toward zero or slightly positive values with increasing duration of opium use, suggesting that any potential beneficial effects on lipid profiles might diminish, or even reverse, over longer periods.

### 3.4 Quality Assessment

**Figure 3:**
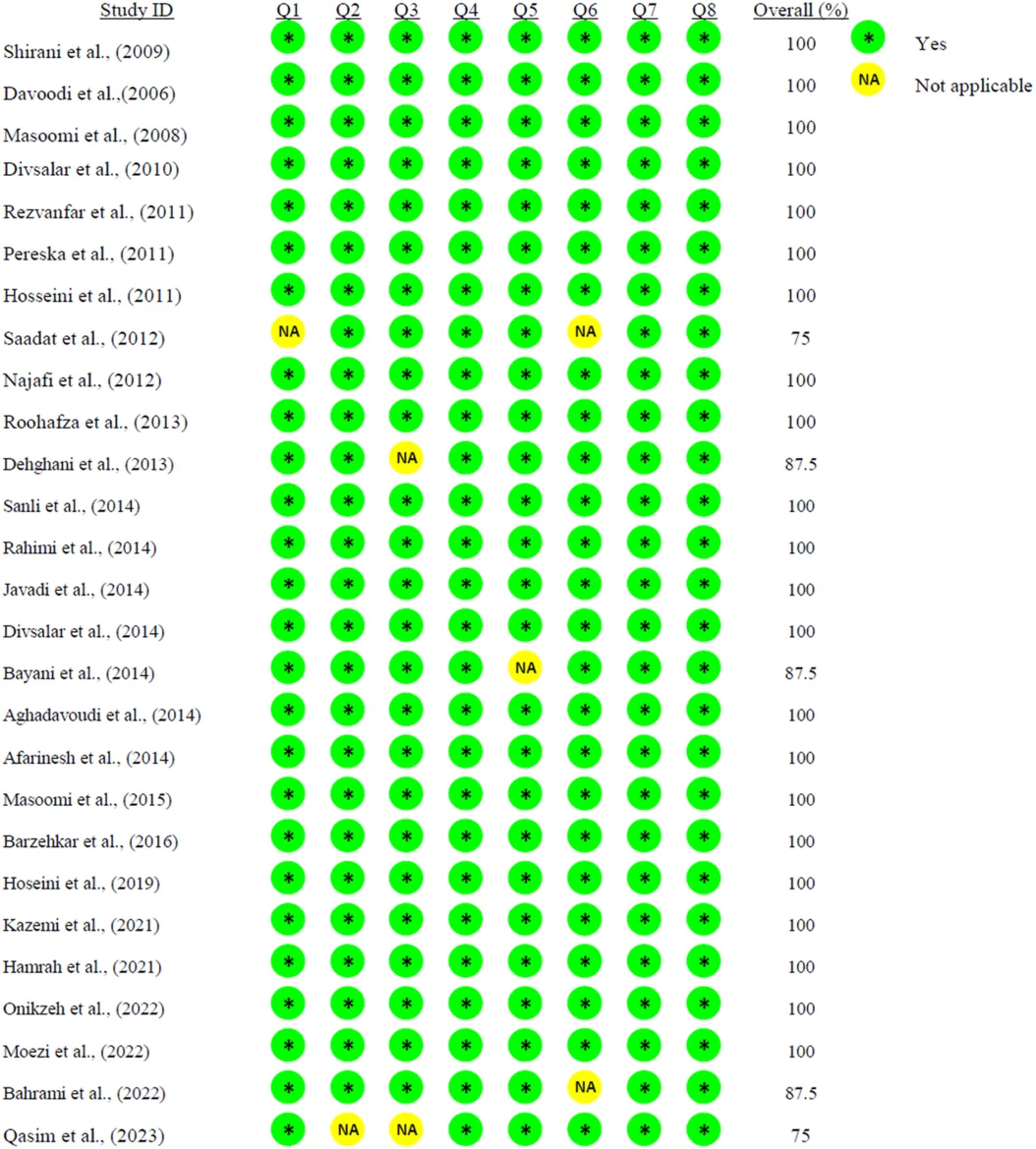
Quality assessment of included articles (cross‐sectional) based on JBI critical appraisal checklist. Q1: Was the exposure measured in a valid and reliable way?, Q2: Were confounding factors identified?, Q3: Were strategies to deal with confounding factors stated?, Q4: Were the outcomes measured in a valid and reliable way? Q5: Was appropriate statistical analysis used?, Q6: Were the study subjects and the setting described in detail?, Q7: Were objective, standard criteria used for measurement of the condition?, and Q8: Were the criteria for inclusion in the sample clearly defined?.

Figure 3 presents the results of the quality assessment for the included cross‐sectional studies, utilizing the JBI Critical Appraisal Tool for Cross‐Sectional Studies. The assessment evaluated each study against eight specific quality criteria (Q1–Q8). The quality assessment revealed a generally high methodological quality across the included cross‐sectional studies. The vast majority of studies, comprising 22 out of 27 (81%), achieved an “Overall (%)” score of 100%, indicating that they fully met all applicable JBI quality criteria. A smaller proportion of studies (3 out of 27, or 11%) scored 87.5%. Only two studies, Saadat et al. [36] and Qasimi et al. [7], scored 75%.

**Figure 4:**
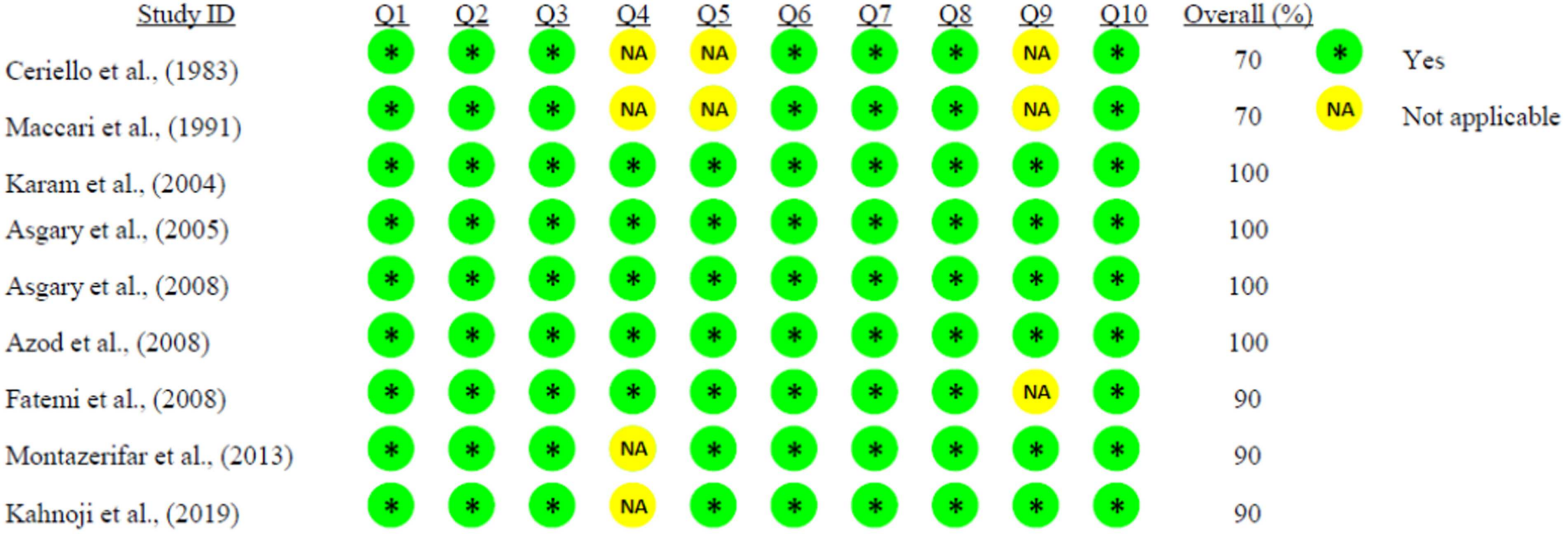
Quality assessment of included articles (case‐control) based on JBI critical appraisal checklist. Q1: Were the groups comparable other than the presence of disease in cases or the absence of disease in controls?, Q2: Were cases and controls matched appropriately?, Q3: Were the same criteria used for identification of cases and controls?, Q4: Was exposure measured in a standard, valid and reliable way?, Q5: Was exposure measured in the same way for cases and controls?, Q6: Were confounding factors identified?, Q7: Were strategies to deal with confounding factors stated?, Q8: Were outcomes assessed in a standard, valid and reliable way for cases and controls?, Q9: Was the exposure period of interest long enough to be meaningful?, Q10: Was appropriate statistical analysis used?.

The provided Figure 4 presents the results of the quality assessment for the included case‐control studies, conducted using the JBI Critical Appraisal Tool for Case‐Control Studies. This assessment evaluated each study against 10 specific quality criteria (Q1–Q10). The quality assessment reveals a generally high methodological quality across the included case‐control studies. A significant proportion of the studies, specifically four out of nine (44%), achieved an “Overall (%)” score of 100%. The remaining studies also exhibited commendable quality, with the majority scoring 90% or 70%. Three studies scored 90%. Lastly, two studies, Maccari et al. [49] and Ceriello et al. [50], scored 70%.

### 3.5 Sensitivity Analysis and Publication Bias

To assess the robustness of our findings and explore potential sources of bias, we conducted comprehensive sensitivity analyses and evaluated for publication bias. Sensitivity analyses involved systematically rerunning the meta‐analysis after excluding individual studies or specific subgroups to ascertain their influence on the overall effect estimates. Across all primary outcomes, the results remained consistent, indicating that no single study or predefined subgroup disproportionately impacted the pooled effect sizes. This consistency strengthens the confidence in the stability and reliability of our main findings. Furthermore, we rigorously assessed for the presence of publication bias using established statistical methods, specifically Egger's regression test and visual inspection of funnel plots. For all outcomes, Egger's test yielded nonsignificant *p* values, suggesting no statistically significant evidence of publication bias. Figure S1 depicts the Egger publication bias plot.

## 4 Discussion

### 4.1 Main Findings

To the best of our knowledge, this is the first systematic review and meta‐analysis to comprehensively examine the effects of opium use on lipid profiles. The findings of this comprehensive meta‐analysis, which included 36 studies with 5660 cases and 11,885 controls, present a complex picture of the impact of opium consumption on lipid metabolism. The main results indicate that while opium use was associated with nonsignificant reductions in TG (WMD = −2.79) and Apo‐A1 (WMD = −24.27), as well as nonsignificant increases in Apo‐B100 (WMD = 3.15) and LDL‐C (WMD = 0.79), there was a statistically significant reduction in TC levels (WMD = −4.72) and HDL‐C (WMD = −1.95). These findings are consistent with the study by Karam and colleagues, which suggested that opium use may exert diverse effects on lipid metabolism [47]. However, the high degree of heterogeneity among the included studies (*I* ^2^ = 79.6% to 96.1%) highlights substantial variability and underscores the necessity of conducting detailed subgroup analyses.

One of the most notable findings of this study is the gender‐based differences in response to opium consumption, which hold significant clinical and scientific implications. In men, a statistically significant reduction in triglyceride levels was observed (WMD = −7.72, *p* = 0.027), whereas this effect was not seen in women. Conversely, women exhibited a significant reduction in TC (WMD = −14.20) and LDL‐C levels (WMD = −10.47), suggesting that opium may exert sex‐specific effects on lipid metabolism. These gender differences may be attributed to variations in metabolic, hormonal, and enzymatic pathways between males and females [51]. Hser and colleagues also reported that men and women respond differently to drug use, which may stem from differences in endocrine function, hepatic metabolism, and adipose tissue distribution [52].

The significant reduction in TC associated with opium use, particularly in the subgroups of women and individuals > 50 years of age, is a noteworthy finding of this study. This observation may be attributed to physiological, hormonal, and metabolic differences within these groups. In women—especially postmenopausal—the decline in estrogen levels may enhance metabolic sensitivity to external factors such as opioids [53]. Similarly, in older individuals, changes in hepatic enzyme activity, a reduced metabolic rate, and a higher burden of underlying diseases may modulate lipid levels in response to opium exposure [54].

The observed effects on lipid profile can be explained by several complex biological mechanisms, the understanding of which is essential for proper interpretation of the findings. The first mechanism involves the impact of opium on the endocrine system, specifically through its influence on the hypothalamic–pituitary–adrenal axis, which can alter the secretion of metabolic hormones [55]. Vuong et al. [56] demonstrated that chronic drug use can lead to alterations in cortisol and thyroid hormone levels, ultimately affecting lipid metabolism [56]. The second mechanism pertains to the influence on hepatic enzymes involved in lipid synthesis and metabolism. Rouach et al. [57] reported that drug use may alter the activity of HMG‐CoA reductase and other key enzymes in the cholesterol biosynthesis pathway. The third mechanism involves the autonomic nervous system, which may be affected by opium through its interaction with opioid receptors [2]. Saltiel [58] showed that activation of opioid receptors can influence insulin signaling as well as glucose and lipid metabolism [58].

Comparison of the results of this meta‐analysis with previous studies reveals noteworthy inconsistencies that may reflect the complexity of the subject. Divsalar and colleagues, in a study conducted on an Iranian population, reported that opium use was associated with reductions in TC and LDL‐C levels, which aligns with our findings in the female subgroup [10]. In contrast, Karam et al. [47] reported that opium consumption was associated with an increase in triglyceride levels, which differs from our results [47]. These discrepancies may be attributed to various factors, including differences in the studied populations, dosage and duration of opium use, measurement methods, and control of confounding variables. Moreover, DesMeules et al. [59] suggested that heterogeneity in meta‐analyses involving drug use is often related to cultural, social, and methodological differences across studies.

The results of the meta‐regression analysis reveal another important dimension of this study, pertaining to the impact of duration of opium use on the lipid profile. Although no statistically significant linear relationship was observed between the duration of opium consumption and changes in lipid parameters, the observed trend suggests that any potentially beneficial effects on lipid profile may diminish or even reverse over time. This finding is consistent with the study by Hedayati‐Moghadam et al. [60], which indicated that the chronic effects of drug use are often different from the acute effects and may change over time. This insight holds clinical significance, as it highlights that the evaluation of opium's effects should not be based solely on short‐term outcomes.

The findings of this study carry significant clinical and public health implications that should be considered in public health decision‐making. From a risk‐benefit assessment perspective, although some potentially positive effects on the lipid profile were observed, these effects can never outweigh the well‐established risks associated with opium use. Degenhardt et al. [61] demonstrated that drug use is associated with a substantial increase in mortality, cardiovascular disease, infections, and other health complications. The observed gender differences further highlight the need for a personalized approach in the evaluation and treatment of individuals who use opium, emphasizing the importance for clinicians to take these differences into account.

### 4.2 Limitations

This study also has several limitations that must be considered when interpreting the results and highlight the importance of caution in drawing conclusions. The cross‐sectional nature of most included studies limits causal inference, as it is not possible to determine with certainty whether the observed changes in lipid profiles are directly caused by opium use or influenced by other factors. Moreover, variation in the definition of opium use across studies—including differences in dosage, duration, and mode of consumption—makes it challenging to compare findings. Inadequate control for confounding factors in some studies is another important issue, as several did not adequately account for critical variables such as diet, physical activity, BMI, and other lifestyle factors, as well as the use of medications that may substantially affect lipid profiles (e.g., lipid‐lowering agents, antidiabetic drugs, and cardiovascular medications). This limitation is particularly relevant among individuals with comorbid conditions such as cardiovascular disease and diabetes, where pharmacological treatment is common and may confound the observed associations. Additionally, the focus on a specific population (with most studies conducted in Iran) restricts the generalizability of the findings to other populations. These limitations underscore the need for well‐designed longitudinal cohort studies with rigorous control of confounding variables, including medication use, to better understand the long‐term health effects of opium use.

## 5 Conclusion

This systematic meta‐analysis, encompassing 36 studies with 5660 cases and 11,885 controls, presents a comprehensive evaluation of the associations between opium consumption and lipid profiles and reveals a complex and heterogeneous pattern of findings. The results indicate that opium consumption is not significantly associated with overall modulation of lipid profiles, and meta‐regression analyses did not demonstrate statistically significant associations between duration of opium use and lipid parameters, including HDL‐C and LDL‐C, despite observing nonsignificant increasing trends over time. Therefore, these findings should not be interpreted as evidence of a definitive adverse or protective effect. While several biological mechanisms—such as interactions with the endocrine system, hepatic enzymes, and opioid receptors—have been proposed in the literature, the current evidence does not support a clear causal or detrimental long‐term lipid effect of opium use. Nevertheless, notable limitations—such as the cross‐sectional nature of most included studies, inadequate control of confounding variables, and limited generalizability due to geographic concentration—must be acknowledged. The observed gender‐based differences highlight potential effect modification and underscore the need for a personalized approach in evaluating opium users. Overall, these findings emphasize the need for well‐designed longitudinal cohort studies with rigorous adjustment for confounders and multinational representation to better elucidate the long‐term cardiometabolic associations of opium consumption.

## Author Contributions

**Fatemeh Zahra Gafuori Fard:** conceptualization, methodology, data curation, formal analysis, writing – original draft, writing – review and editing. **Yaser Mohammadi:** validation, formal analysis, visualization, writing – original draft, writing – review and editing. **Maryam Rezaei:** investigation, resources, data curation, writing – review and editing. **Seyed Mohammad Riahi:** supervision, project administration, methodology, writing – review and editing, funding acquisition.

## Disclosure

Seyed Mohammad Riahi, as the corresponding author and manuscript guarantor, affirms that this manuscript is an honest, accurate, and transparent account of the study being reported; that no important aspects of the study have been omitted; and that any discrepancies from the study as planned (and, if relevant, registered) have been fully explained.

## Ethics Statement

This study was approved by the Ethics Committee of Birjand University of Medical Sciences, under the approval code IR.BUMS.REC.1403.313.

## Conflicts of Interest

The authors declare no conflicts of interest.

## Supporting information


Supporting File

## Data Availability

Data will be made available on request.
